# Cytotoxic polyfunctionality maturation of cytomegalovirus-pp65-specific CD4 + and CD8 + T-cell responses in older adults positively correlates with response size

**DOI:** 10.1038/srep19227

**Published:** 2016-01-18

**Authors:** Yen-Ling Chiu, Chung-Hao Lin, Bo-Yi Sung, Yi-Fang Chuang, Jonathan P. Schneck, Florian Kern, Graham Pawelec, George C. Wang

**Affiliations:** 1 Institute of Cell Engineering, Johns Hopkins School of Medicine, USA; 2 Department of Medicine and Nephrology, Far Eastern Memorial Hospital, Taiwan; 3 Graduate Program of Biomedical Informatics, Yuan Ze University College of Informatics, Taiwan; 4 Division of General Medicine and Geriatric Medicine, Chang Gung Memorial Hospital at Linkou, Chang Gung University College of Medicine, Taiwan; 5 Department of Microbiology and Immunology, National Defense Medical Center, Taiwan; 6 Department of Epidemiology, National Yang Ming University School of Public Health, Taiwan; 7 Division of Medicine, Pathogen Host Interaction, Brighton and Sussex Medical School, United Kingdom; 8 Department of Internal Medicine II, University of Tubingen Center for Medical Research, Germany; 9 Division of Geriatric Medicine and Gerontology, Biology of Healthy Aging Program, Johns Hopkins University School of Medicine, USA

## Abstract

Cytomegalovirus (CMV) infection is one of the most common persistent viral infections in humans worldwide and is epidemiologically associated with many adverse health consequences during aging. Previous studies yielded conflicting results regarding whether large, CMV-specific T-cell expansions maintain their function during human aging. In the current study, we examined the *in vitro* CMV-pp65-reactive T-cell response by comprehensively studying five effector functions (i.e., interleukin-2, tumor necrosis factor-α, interferon-γ, perforin, and CD107a expression) in 76 seropositive individuals aged 70 years or older. Two data-driven, polyfunctionality panels (IL-2-associated and cytotoxicity-associated) derived from effector function co-expression patterns were used to analyze the results. We found that, CMV-pp65-reactive CD8 + and CD4 + T cells contained similar polyfunctional subsets, and the level of polyfunctionality was related to the size of antigen-specific response. In both CD8 + and CD4 + cells, polyfunctional cells with high cytotoxic potential accounted for a larger proportion of the total response as the total response size increased. Notably, a higher serum CMV-IgG level was positively associated with a larger T-cell response size and a higher level of cytotoxic polyfunctionality. These findings indicate that CMV-pp65-specific CD4 + and CD8 + T cell undergo simultaneous cytotoxic polyfunctionality maturation during aging.

Human cytomegalovirus (CMV) is a common beta human herpesvirus with an estimated infection prevalence of more than 50% of the world population^1^. After primary infection, which frequently occurs during early childhood, CMV establishes lifelong latency. While CMV was originally thought to be a harmless viral infection in immunocompetent individuals, others and we have shown that CMV seropositivity is in fact associated with many adverse consequences during normal aging^2^,^3^,^4^. For example, it is associated with an increased risk for hypertension^5^, cardiovascular diseases^6^,^7^ and mortality^4^,^8^,^9^,^10^ and considered by some to be a causative agent. CMV infection may also be associated with unresponsiveness to influenza vaccination^11^.

Both CD4 + and CD8 + T cells are required to control CMV infection^1^,^12^,^13^. While a healthy immune system is usually able to contain CMV and prevent it from causing overt clinical diseases (although CMV-reactivations causing mild symptoms may often be overlooked or not identified as caused by CMV), over time the virus acts as an enormous burden on the immune system. It is estimated that about 9-10% of the human memory T-cell compartment recognizes CMV-derived epitopes^14^. The number can be dramatically higher in the elderly^15^,^16^,^17^, possibly resulting from chronic antigenic stimulation caused by intermittent, subclinical reactivations of the virus throughout the lifetime. Such an accumulation of CMV-specific memory T cells may be maintained through a continuous replacement of short-lived, functional T cells^18^ and/or accumulation of apoptosis-resistant late-stage differentiated or “senescent” T cells^19^.

In many infectious diseases, immunological control of pathogens including CMV has been associated with the emergence of polyfunctional T cells capable of executing multiple effector functions^20^,^21^,^22^,^23^. In contrast, less-polyfunctional, or even “exhausted” T cells may dominate immune responses during chronic infections, such as those by human immunodeficiency virus^24^ and hepatitis C virus^25^. These T cells are characterized by a progressive loss of effector functions and, hence, loss of polyfunctionality, coupled with clonal expansion, and possibly replicative senescence^26^. Similarly, CMV-specific T cells undergo significant clonal expansion, especially in older adults^16^,^27^,^28^. It has been suggested that clonal expansion of CMV-specific T cells in the elderly negatively impacts on their functionality, as a limited number of studies enrolling older adults^17^,^19^ showed that a greater proportion of CMV-pp65-specific T cells do not produce IFNγ in response to antigen stimulation. However, only one effector function was analyzed in these studies and potential variations in polyfunctionality among individuals with varying degrees of clonal expansion was not studied. In contrast, study performed in aged rhesus macaques showed that CMV-specific immunity is maintained and the response to and protection against an *in vivo* CMV challenge was identical in adult and aged macaques^29^. A recent human study^30^ performed in a cohort of diverse age showed that CMV-specific total response size positively correlated the frequencies of certain polyfunctional subsets. Nevertheless, the study included few older adults and the polyfunctionality markers were limited. It lacked important cytotoxicity measurements, in particular perforin and CD107a. It remains unclear whether any functional T-cell subset would preferentially expand in large CMV-specific responses, and whether CD4 + and CD8 + T cells undergo similar changes.

To answer these questions and gain further insight into the polyfunctional profiles of CMV-specific T cells during aging, we studied a comprehensive CMV-pp65-specific polyfunctionality signature in a cohort of adults aged 70 years or older. CMV-pp65 is an immunodominant protein that has a large impact on the T-cell repertoire in CMV-seropositive individuals^31^,^32^. In the present study, we defined CMV-pp65-specific polyfunctional responses by simultaneously measuring interleukin-2 (IL-2), tumor necrosis factor-α (TNFα), interferon-γ (IFNγ), CD107a, and perforin expression in both CD8 + and CD4 + T cells. Because IL-2 and perforin were found to be mutually exclusive functions, we analyzed polyfunctionality using either an IL-2-associated polyfunctionality panel (co-expression of IL-2, TNFα, and IFNγ) or a cytotoxicity-associated polyfunctionality panel (co-expression of TNFα, IFNγ, CD107a, and perforin). We found that CD4 + and CD8 + CMV-pp65-specific T cells were dominated by the same functional subsets. For both CD8 + and CD4 + CMV responses, a higher degree of cytotoxicity-associated polyfunctionality positively correlated with a larger total CMV-specific response size. In contrast, IL-2-associated polyfunctionality did not follow the same trend. Although IL-2-associated polyfunctionality in CD8 + T cells was lower in large CMV responses, IL-2-associated polyfunctionality in CD4 + T cells was not dependent on response size. Finally, T-cell polyfunctionality and response size were positively correlated with serum CMV-IgG level. These findings suggest that, even in older adults, both CD8 + and CD4 + CMV-pp65-specific T cells do not undergo functional exhaustion in large CMV-specific responses, and the degrees of T-cell polyfunctionality positively correlate with the intensity of the humoral response against CMV.

## Methods

### Study participants

Heparinized venous whole blood was obtained from immunocompetent individuals not taking immunomodulatory pharmaceutical agents. A total of 76 CMV-seropositive elderly donors (55% female, 45% male) were included in this study. The age range was 71 to 93 years (mean age + /−standard deviation, 80 + /−5.7 years). The study protocol conforms to the Declaration of Helsinki and was approved by the institutional review board of Johns Hopkins University, Baltimore, MD. Informed consent was obtained from all participants.

### Measurement of CMV IgG level

CMV IgG concentration was measured in stored (−70 °C) serum specimens using a commercial enzyme-linked immunosorbent assay kit (GenWay Biotech). Seropositivity for CMV was defined according to the manufacturer’s instructions, as a serum IgG concentration of >1.2 IU/ml.

### Cell preparation and peptide pool stimulation

The following protocol is prepared according to the MIATA (**M**inimal **I**nformation **A**bout **T** cell **A**ssays) recommendations (www.miataproject.org). Briefly, peripheral blood mononuclear cells (PBMCs) were collected from each donor’s whole blood by density gradient centrifugation (GE Healthcare Ficoll-Paque PLUS), resuspended in supplemented RPMI media containing 10% DMSO before cryopreserved in liquid nitrogen. Before use for stimulation, frozen PBMCs were thawed quickly in 37 °C, washed twice with complete RPMI medium, and rested overnight in complete RPMI medium containing 10% fetal bovine serum and 10μg/ml DNase I (Sigma).

PBMCs were stimulated at a density of 1 million per 100 μl of complete RPMI medium in a 96-well U bottom plate using a CMV-pp65 peptide pool containing 138 peptides derived from a peptide scan (15mers with 11 amino acids overlap) of the 65kDa phosphoprotein (Swiss Protein ID: P06725) of human CMV. Cells were incubated with the CMV-pp65 peptide pool (1μg/ml per peptide, PepMix, JPT Peptide Technologies), anti-CD28/CD49d (BD Biosciences), anti-CD107a (BD, clone H4A3), Golgistop (monensin, BD), and Golgiplug (Brefeldin A, BD) for six hours at 37 °C.

### Intracellular cytokine assay and polyfunctionality analyses

At the end of stimulation, cells were washed with FACS washing buffer and stained with anti-CD3 BV510 (clone OKT3, Biolegend), anti-CD8 APC-Cy7 (clone RPA-T8, Biolegend), anti-CD4 Pacific Blue (clone SK3, Biolegend) and Live/Dead® cell viability assay (Invitrogen) for 20 minutes before fixation with Cytofix/Cytoperm buffer (BD Biosciences) overnight at 4 °C. On the next day, cells were washed with 1X Perm/Wash solution (BD Biosciences) and stained with anti-perforin-PE (clone B-D48, Biolegend), anti-IL-2-PerCP-Cy5.5 (clone MQ1-17H12, Biolegend), anti-TNFα-PE-Cy7 (clone Mab11, Biolegend), anti-IFNγ-APC (clone 25723.11, BD), at 4 °C for two hours. After washing, cytokine responses were acquired immediately using a BD LSRII flow cytometer.

Flow cytometry results were analyzed using FlowJo (Tree Star). A lymphocyte gate based on FSC/SSC, a singlet gate, and a live/dead cell gate were applied before gating on CD3 + CD8 + and CD3 + CD4 + cells. Subsequently, a population positive for each cytokine/effector function was gated. To obtain co-expression pattern, a combinatorial gating strategy based on the gates of each effector function was applied using the FlowJo Boolean gate platform. After subtracting the background response (with co-stimulation but no peptide antigen), a positive cytokine response was defined as at least 0.03% of CD3 + T cells and at least 40 events^33^. Total CMV-pp65-specific T-cell response was derived by summing the percentages of cells expressing at least one effector function among all CD8 + or CD4 + T cells (%CD8 + or %CD4 + ).

### Statistical analyses

Simplified Presentation of Incredibly Complex Evaluations (SPICE, National Institutes of Health, available at http://www.niaid.nih.gov/) was used to analyze polyfunctionality profiles. A polyfunctionality profile was first created using all five effector functions. To create the IL-2-associated polyfunctionality profile, the ability of cells to upregulate CD107a and perforin was excluded using the “sum” function. Similarly, to create the cytotoxicity-associated polyfunctionality profile, the signal of IL-2 production was excluded. Permutation tests set at 10,000 repetitions were used to test the differences between pie charts. Other statistical analyses were performed using Prism 5 (Graph Pad). For comparison between two groups of samples with parametric distribution (such as cytokine MFI), Student’s t-test was used. For comparison between two groups of samples with non-parametric distribution, non-parametric Mann-Whitney U test was used. Spearman’s rank-order correlation test was used to detect significant correlations between two parameters of interest.

## Results

### Polyfunctional response patterns of CD8 + and CD4 + T cells to CMV-pp65 peptide pools are similar

For the purpose of modeling CMV-specific T-cell polyfunctionality in this study, PBMCs were stimulated with a CMV-pp65 peptide pool and analyzed for intracytoplasmic IL-2, TNFα, IFNγ, CD107a, and perforin expression. IFNγ, TNFα, and CD107a are typical CD8 + effector functions used to characterize virus-specific T-cell function^20^,^24^,^34^. IL-2 is typically expressed in memory T cells in the early differentiation stage. The ability of CMV-specific T cells to produce IL-2 has been implicated in protection against CMV-related disease^23^,^35^. In contrast, perforin is expressed in highly active effector T cells that do not produce IL-2^36^. Upon antigen stimulation, human T cells rapidly upregulate perforin de novo, which is immediately transported to the immunological synapse where it potentiates cytotoxicity^37^,^38^. We adopted the gating strategy reported by Makedonas *et al*^38^ to identify CMV-specific T cells capable of rapid perforin upregulation and used upregulation of perforin as one of the effector functions in this study.

**Figure 1:**
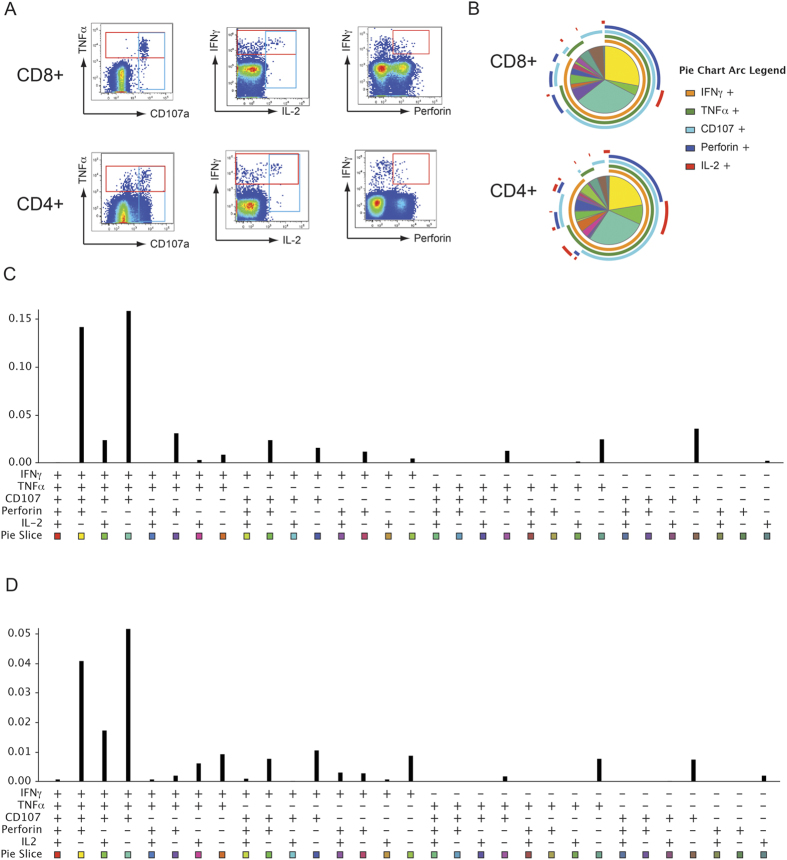
Polyfunctionality profiles of CD8 + and CD4 + CMV-specific T cells in older adults are similar. A comprehensive profile of CMV-pp65-specific T-cell function, including IL-2, TNFα, IFNγ, CD107a, and perforin, was analyzed in a cohort of older donors. **(A)** Representative results of flow cytometry staining of CMV-specific effector functions. The plots are obtained from a donor with moderately sized CD8 + and CD4 + responses. **(B)** Pie charts demonstrating the relative contribution of each of 31 possible functional subsets among the total CD8 + or CD4 + response in all participants. An accompanying bar graph depicting the average frequency of each functional subset among the CD8 + or CD4 + population is shown in Fig. 1C,D. **(C**,**D)** Frequency of each CMV-specific functional subset among CD8 + (C) or CD4 + (D) T cells in all participants. The landscapes of the functional subsets were remarkably similar between CD8 + and CD4 + cells. In both CD8 + and CD4 + T cells, the IFNγ + TNFα + CD107a + perforin + IL-2- subset and the IFNγ + TNFα + CD107a + perforin-IL-2- subset dominated the response.

After background subtraction, 66 of 76 CMV-seropositive individuals (86.8%) showed positive cytokine response. Figure 1A depicts representative flow cytometry staining results from an individual with both CD8 + and CD4 + T-cell responses. Total CMV pp65-specific response size defined as the frequency of cells capable of at least one effector function, was higher for CD8 + than CD4 + T cells (mean value, 0.38% vs. 0.14%). When individual effector functions were analyzed among the total functional responses, the proportion of cells with any cytotoxic function (CD107a or perforin upregulation) was higher among CD8 + than CD4 + T cells (93.7% versus 72.1%, p value = 0.013). In contrast, the proportion of cells producing IL-2 was higher among CD4 + than CD8 + T cells (19% versus 4.6%, p value < 0.001).

When the total response was further delineated by using all 31 possible functional combinations, we found that there were no cells capable of all of five functions assessed (Fig. 1B). This is due to the fact that IL-2 and perforin upregulation were mutually exclusive functions, consistent with findings from an earlier study^36^. The polyfunctionality profile pie chart (showing the relative contribution of each functional subset among the total functional responses) was very similar between CD8 + and CD4 + T cells (Fig. 1B). In addition, the same polyfunctional subsets were dominant in both CD8 + and CD4 + T cells. As shown in Fig. 1C,D, both CD8 + and CD4 + CMV-pp65-specific T cells contained a polyfunctional subset with high cytotoxic potential (IFNγ + TNFα + CD107a + perforin + IL-2- [I + T + C + P + 2- subset]), as well as clearly detectable IFNγ + TNFα + CD107a + perforin-IL2 + (I + T + C + P-2 + ) and IFNγ + TNFα + CD107a + perforin-IL-2- (I + T + C + P-2-) subsets.

### Polyfunctional CMV-pp65-specific cells have enhanced effector functions

**Figure 2:**
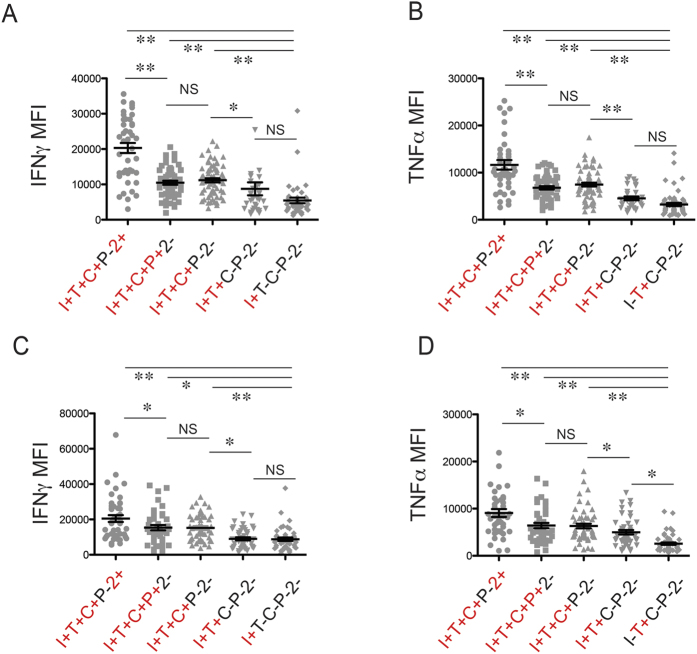
Polyfunctional T cells exhibit enhanced effector functions. Five CMV-pp65-specific functional subsets within CD8 + (**A**,**B**) or CD4 + (**C**,**D**) T cells were compared with respect to their mean fluorescence intensity (MFI) of TNFα and IFNγ staining. I, IFNγ; T, TNFα; C, CD107a; P, perforin; 2, IL-2. **(A,B)** In CD8 + T cells, the I + T + C + P-2 + subset displayed the strongest staining for IFNγ and TNFα when compared with the I + T + C + P + 2- and the I + T + C + P-2- subset. The dual functional, I + T + C-P-2- subset and the monofunctional, I + T-C-P-2- or I-T + C-P-2- subset produced even lower levels of IFNγ and TNFα. **(C,D)** Higher MFIs for IFNγ and TNFα in polyfunctional subsets were also observed in CMV-specific CD4 + T cells. The comparisons were carried out individually by paired Student’s t-test. The threshold for statistical significance was corrected following the Bonferroni method. NS: non-significant; *p value < 0.007; **p value < 0.0007.

While highly polyfunctional CMV-specific T cells are by definition capable of more types of effector functions than less polyfunctional cells, it has also been shown that the former perform a given function as well as or superior to the latter^22^. Nevertheless, numerical differences between polyfunctionality levels (for example, three functions versus four functions) are still misleading in their specificity and unclear in their biological meaning. We thus looked at the expression level of IFNγ and TNFα in pp65-specific T cells within different polyfunctional subsets (Fig. 2). Clearly, the I + T + C + P-2 + subset in either CD8 + or CD4 + cells produced the highest level of IFNγ or TNFα. While also being four-function capable, the I + T + C + P + 2- subset produced fewer effector cytokines on a per-cell basis than the I + T + C + P-2 + subset, with a level similar to the tri-functional I + T + C + P-2- subset. Bi-functional and monofunctional cells have even lower expression levels of IFNγ and TNFα. In summary, while polyfunctional T cells in general have enhanced cytokine functions compared with oligofunctional T cells, cytokine expression levels differ among T-cell subsets capable of the same number of functions, such as the four-functional I + T + C + P-2 + and the four-functional I + T + C + P + 2- subsets.

### IL-2-associated polyfunctionality and cytotoxicity-associated polyfunctionality among CMV-pp65-specific CD8 + T cells

**Figure 3:**
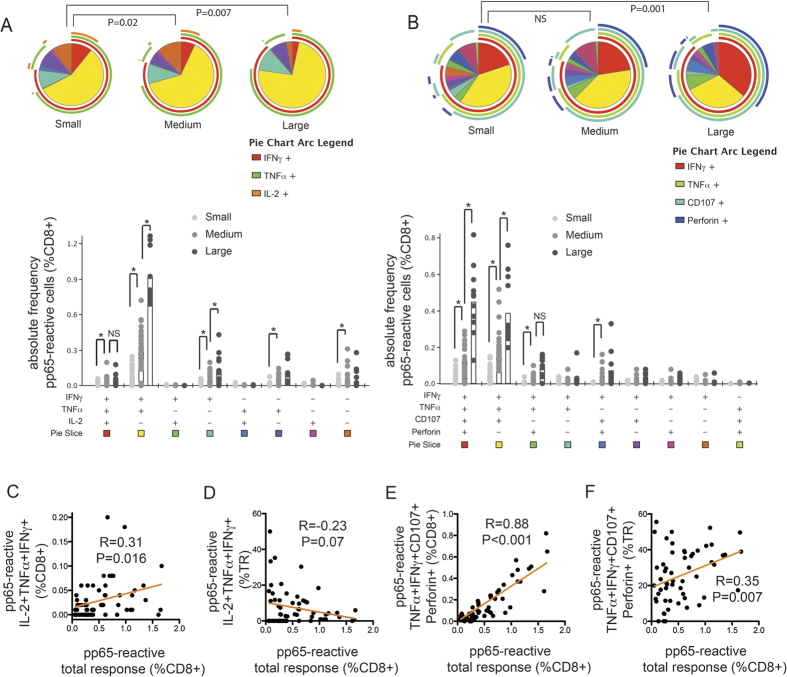
The size of the total CMV-specific CD8 + T-cell response determines its polyfunctionality profile. Based on the total frequency (%CD8+) of CMV-reactive cells among CD8 + T cells (or total response, TR), participants were separated into three response-size groups: large, >1%; medium, 0.5–1%; and small, <0.5%. Polyfunctionality was analyzed according to two different combinations (panels) of cellular expression. The first panel consisted of three cytokine effector functions associated with memory T cells: IL-2 (2), TNFα (T), and IFNγ (I) (Panel A). The second panel consisted of four effector functions associated with the cytotoxic effector phenotype: perforin (P), CD107a (C), TNFα (T) and IFNγ (I) (Panel B). Results are summarized using pie charts (showing the relative contribution of each functional subset among the total response) and bar graphs (showing the frequency of each functional subset within total CD8 + cells, %CD8). Each color slice of the pie chart corresponds to a specific functional subset shown in the corresponding bar graph. For simplicity, some subsets without any cells are hidden from the bar graphs. **(A)** Large, medium, and small TRs harbored different levels of IL-2-associated polyfunctionality. The I + T + 2 + subset represented a smaller proportion of the TR (shown as the red slice in the pie charts) in the large than smaller TRs. However, the frequency of the I + T + 2 + subset within total CD8 + T cells (%CD8) was higher in participants with larger TRs. **(B)** Different TR sizes harbored different levels of cytotoxic polyfunctionality. The I + T + C + P + subset constituted a larger proportion of the large TR than it did in the small TR. The large TR at the same time also comprised a higher frequency of I + T + C + P + cells within total CD8 + T cells (%CD8) (shown as the red slice in pie charts). **(C)** Linear regression analysis showed that the I + T + 2 + subset frequency (%CD8) positively correlated with TR size. **(D)** However, the proportion of I + T + 2 + subset within TR negatively correlated with TR size. **(E,F)** Both the I + T + C + P + subset frequency (%CD8) and its proportion within TR positively correlated with TR size.

Because some previous studies suggested that the CD8 + CMV-specific T-cells accumulating in the elderly were in some respects dysfunctional^19^,^27^, we asked whether CMV-specific T cells would differ in the degree of polyfunctionality among individuals with different total CMV-specific response sizes. As IL-2 and perforin expression is mutually exclusive, we investigated the change in polyfunctionality in relation to total response size according to two different combinations (panels) of cellular expression: IL-2-associated polyfunctionality was analyzed using the functional combination of IL-2, IFNγ, and TNFα expression (Fig. 3A), while cytotoxicity-based polyfunctionality was analyzed using the functional combination of IFNγ, TNFα, CD107a, and perforin expression (Fig. 3B). Besides the polyfunctionality profile, which portrays the relative contribution of each T-cell functional subset, we also focused on the frequency of the most polyfunctional T-cell subset in each panel: the I + T + 2 + subset and the I + T + C + P + subset, for the subsequent analyses.

We first separated the participants into three groups based on their total CMV-specific CD8 + T-cell response size (%CD8 + ) defined as follows: large, >1%; medium, 0.5-1%; and small, <0.5%. As shown in the bar graphs in Fig. 3A and Fig. 3C, the frequency of CMV-pp65-specific I + T + 2 + polyfunctional CD8 + T cells (%CD8 + ) increased as the total CMV-pp65-specific CD8 + T-cell response size increased. The relationship between the frequency of pp65-specific I + T + C + P + polyfunctional CD8 + T cells and total pp65-specific CD8 + T-cell response size also followed the same trend (bar graphs in Fig. 3B and Fig. 3E). These findings suggest that expansions of both of these polyfunctional CD8 + T-cell subsets occurred as total response size increased.

Although both polyfunctional CD8 + T-cell subsets accumulated (increased their frequencies among CD8 + T cells) in relation to increased total response size, the CD8 + T-cell polyfunctionality profile, reflecting the relative contribution of each polyfunctional subset to the total response, is significantly different among different total response sizes. Specifically, as shown in Fig. 3A (pie charts) and Fig. 3D, the proportions of the CD8 + , I + T + 2 + T-cell subset among total responses significantly decreased as total response size increased. In contrast, the proportion of the CD8 + , cytotoxic I + T + C + P + T-cell polyfunctional subset increased as total response size increased (Fig. 3B pie charts, Fig. 3F). Thus, depending on the polyfunctionality panel being considered, the pp65-specific CD8 + T-cell response can be either relatively more polyfunctional (cytotoxicity response) or less polyfunctional (IL-2-associated response) with larger total response size.

### IL-2-associated polyfunctionality and cytotoxicity-associated polyfunctionality among CMV-pp65-specific CD4 + T cells

**Figure 4:**
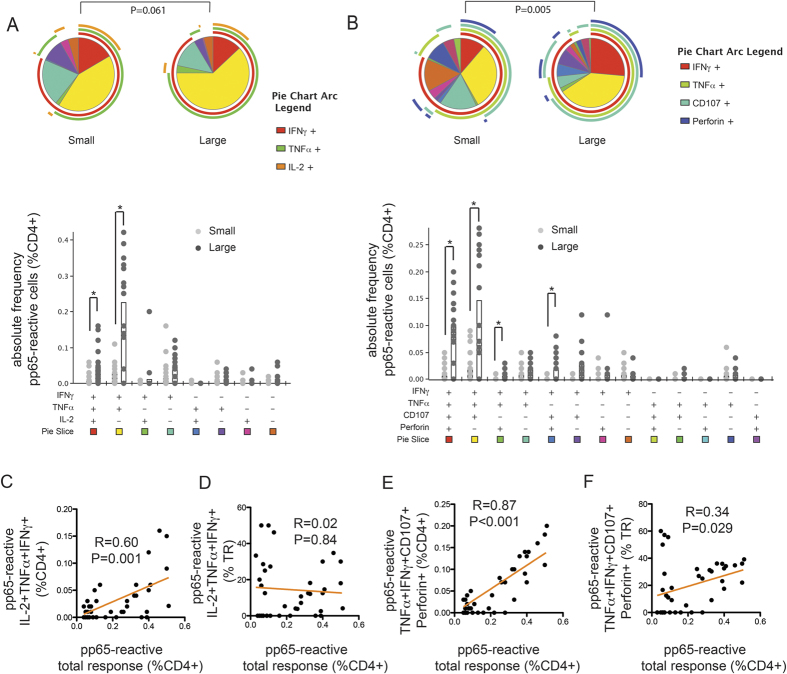
Size of the CMV-specific CD4 + T-cell response determines cytotoxic but not IL-2-associated polyfunctionality profile. Based on the total frequency (%CD4 + ) of CMV-reactive cells among CD4 + T cells (or total response, TR), participants were separated into either the large (>0.3%) or small (<0.3%) TR group. Polyfunctionality was analyzed as described in Fig. 3. The first panel consisted of three cytokine effector functions associated with young memory T cells: IL-2 (2), TNFα (T), and IFNγ (I) (Panel A). The second panel consisted of four effector functions associated with the cytotoxic effector phenotype: perforin (P), CD107a (C), TNFα (T) and IFNγ (I) (Panel B). **(A)** Large and small TRs harbored similar IL-2-associated polyfunctionality. The I + T + 2 + subset represented a non-significantly smaller proportion of total TR (shown as the red slice in the pie charts) in large than small TR. However, the frequency of the I + T + 2 + subset within total CD4 + T cells (%CD4) was higher in participants with large than small TR. **(B)** Different TR sizes harbored different levels of cytotoxic polyfunctionality. Large TR contained a higher proportion of the I + T + C + P + subset than small TR. Individuals with large TR also had a higher frequency of I + T + C + P + cells (%CD4) (shown as the red slice in the pie charts). **(C)** Linear regression analysis showed that the I + T + 2 + subset frequency (%CD4) positively correlated with TR size. **(D)** Consistent with the observation in (A), the proportion of the I + T + 2 + subset was not correlated with TR size. **(E,F)** Both the I + T + C + P + subset frequency (%CD4) and its proportion within TR positively correlated with TR size. Overall, the results suggest that the expansion of CMV-specific CD4 + T cells is associated with progressive cytotoxic effector function maturation.

We also performed similar polyfunctionality analyses on CD4 + T cells (Fig. 4). Samples were separated into two groups instead of three based on CD4 + T-cell response size because the overall response size was smaller for CD4 + T cells: large, >0.3%; and small, <0.3%. Overall, compared with CMV-pp65-specific CD8 + T-cell responses, CMV-pp65-specific CD4 + T cells had a higher proportion of cells capable of IL-2 expression and a lower proportion capable of cytotoxic functions. Consistent with the observations made for the CD8 + T-cell population, the frequencies of both I + T + 2 + and the cytotoxic I + T + C + P + populations were higher in large CD4 + T cell responses, the latter also representing a larger proportion of the activated cells. However, unlike in CD8 + T cells, the proportion of the I + T + 2 + subset was the same in large and small CD4 + T cell responses. In summary, both CD8 + and CD4 + pp65-specific T cells undergo progressive cytotoxicity-associated polyfunctionality maturation in older adults with expanding CMV-specific responses.

### Relationships among pp65-specific T-cell response size, polyfunctionality, and serum CMV IgG level

It is known that CMV-specific T-cell response size is highly variable among individuals. Previous studies have shown that certain HLA types can affect pathogen-specific T-cell frequency and functions^39^,^40^. In addition, a large response size could also result from more frequent viral reactivations *in vivo* and thus greater expansion of antigen-specific cells. To better understand the relationships between the CMV-specific humoral response *in vivo* and the CD4 + and CD8 + cellular response *in vitro*, we first examined the relationship between CMV-pp65-specific CD8 + and CD4 + T-cell responses. We found that the total pp65-specific CD8 + and CD4 + T-cell responses were positively correlated (R = 0.347, p value = 0.0053; Table 1). Further, both pp65-specific CD8 + and CD4 + total response sizes were positively correlated with serum CMV IgG level. Because CMV IgG antibody titer has been found to increase linearly with the level of viremia and viral load in leukocytes^41^,^42^, the current findings support the notion that a large CMV-specific T-cell response size reflects more frequent and/or more severe viral reactivations in these older individuals.

**Figure 5:**
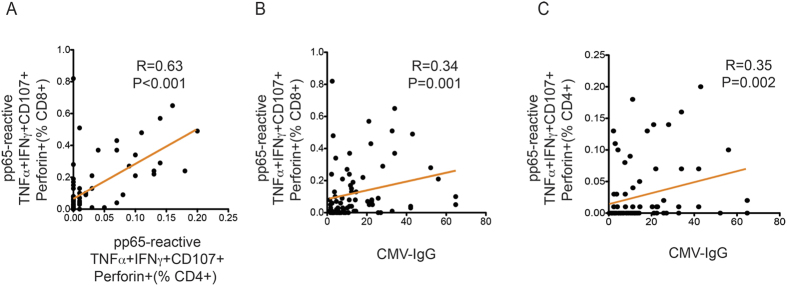
Cytotoxic T-cell polyfunctionality positively correlates with CMV-IgG level. **(A)** Cytotoxic CD4 + T-cell polyfunctionality positively correlated with cytotoxic CD8 + T-cell polyfunctionality. **(B,C)** Level of CMV-IgG positively correlated with CD8 + and CD4 + T-cell cytotoxic polyfunctionality. Spearman’s rank-order correlation test was used to detect significant correlations between two parameters of interest.

Given the significant correlation between total response size and polyfunctionality (Figs 3 and 4), we also examined the relationship between polyfunctionality and total response size across CD4 + and CD8 + T-cell responses. Interestingly, we found that the frequency of CD8 + cytotoxic polyfunctional T cells (%CD8 + , T + I + C + P + ) was positively correlated with the total CD4 + T-cell response size, while the frequency of CD4 + IL-2-associated polyfunctional T cells (%CD4 + , I + T + 2 + ) was positively correlated with the total CD8 + response size. Notably, the degree of CD8 + cytotoxic polyfunctionality positively correlated with CD4 + T-cell polyfunctionality and the level of CMV IgG positively correlated with the level of CD4 + and CD8 + cytotoxic polyfunctionality (Fig. 5). Overall, these results suggest that the humoral response is closely related to the clonal expansion and cytotoxic polyfunctionality maturation of both CD4 + and CD8 + CMV-specific T cells in older adults.

## Discussion

Some published reports have associated the accumulation of CMV-specific, late-differentiated memory cells in older adults with impaired immune competence^11^,^43^,^44^ and argued that these contribute to the immunosenescent phenotype as well as the immune risk profile (IRP)^2^,^3^,^45^. Others, however, have reported that highly polyfunctional T cell subsets accumulate in such expanded responses^30^. While older adults are at higher risk of experiencing many infection-related illnesses, it is not yet clear whether established anti-viral immunity remains robust in older age or gradually becomes exhausted. Indeed, recent evidence suggests that virus-specific T cell polyfunctionality does not decrease with age^46^. However, limited longitudinal studies have associated a loss of predominantly CMV-specific CD8 + T cell clonal expansions with incipient mortality in the very elderly^27^. In order to gain better insight into how the immune system controls CMV infection and how this may contribute to the immunosenescent phenotype, further studies are required.

In the current study, we performed a comprehensive analysis of the CMV-pp65-reactive polyfunctionality landscape in older adults by simultaneously measuring five T-cell effector functions and analyzing the results according to two combinations of cytokine co-expression pattern. To further build on findings from previous studies, we adopted a unique, data-driven approach to analyzing the complete landscape of anti-CMV-pp65 T-cell polyfunctionality response. Because IL-2 and perforin were mutually exclusive functions, we analyzed the polyfunctionality profile by two different panels. We demonstrated that in older adults, both CMV-pp65-specific CD4 + and CD8 + T-cell responses were dominated by the same functional subsets, i.e., T cells simultaneously expressing TNFα, IFNγ, and CD107a but not IL-2 or perforin, and T cells simultaneously expressing TNFα, IFNγ, CD107a, and perforin but not IL-2. Most importantly, we showed that CMV-pp65-specific T cells in older adults did not undergo functional exhaustion even in large responses. Instead, these cells exhibited evidence of greater cytotoxicity-associated polyfunctionality maturation into an “effector”-like phenotype in larger response sizes. The frequency (%CD8 + or %CD4 + ) of TNFα + IFNγ + CD107a + perforin + IL2- polyfunctional T cells was higher among larger total response sizes, in both CD8 + and CD4 + populations. Clearly, polyfunctional cells were not superseded by less polyfunctional cells in large CMV-specific T-cell responses, even in the elderly. These findings appear to refute those of several previous studies^19^,^27^, which suggested that clonally expanded CMV-specific T cells are to some extent dysfunctional in older adults. However, in those studies, CMV-specific T-cell function was only analyzed based on a combination of tetramer staining and IFNγ production, without measuring other effector functions. In our study, T cell function was probed without tetramer staining, thus direct comparison between studies is not possible. Although we can not exclude the possibility that certain pp65-specific cells may not have any effector function thus will not be detected by our assay, using tetramers to identify antigen-specific cells will only narrow the repertoire of response in general we are interested in. Furthermore, we believe the combination of tetramer staining along with peptide stimulation potentially could be biased because tetramers inevitably compete with the added peptide for T-cell receptor binding, and the measured level of polyfunctionality could be severely affected. Nevertheless, in future studies, it maybe possible to use certain activation markers such as CD137, CD69 and CD40L to identify antigen-specific cells.

The observation that polyfunctional cytotoxic T cells comprised a dominant population in both CD8 + and CD4 + CMV-pp65-specific T cells suggests that the expansion of polyfunctional cytotoxic T cells is the driving force for large response size in T-cell immunity against CMV. A larger proportion of cytotoxic polyfunctional cells among CMV-specific T cells was associated with a reciprocally smaller proportion of IL-2-producing polyfunctional cells among CMV-specific T cells in CD8 + T-cell but not in CD4 + T-cell responses. Because IL-2 production is a feature of naïve and central memory cells and is the first function to be downregulated during progression towards a more differentiated phenotype^47^,^48^, pp65-specific CD4 + T cells may be less differentiated than their CD8 + counterparts. IL-2-producing CMV-specific T cells are implicated in preventing CMV-related clinical disease, including in transplant recipients^23^,^49^ and in HIV-infected patients^35^, and thus may also protect older adults from CMV-related acute illness. Our results showed that the frequencies of these cells within the total CD4 + and CD8 + T-cell populations did not decline but, rather, increased in individuals with larger CMV-specific responses. Such findings might explain why overt CMV-related acute illnesses are rare even in older adults and could represent a compensatory mechanism to maintain CMV immunosurveillance. This interpretation is consistent with the earlier observations that although the frequency of functional IFNγ-producing CD8 + T cells carrying receptors for a CMV-pp65 epitope was lower in the elderly than the young, the accumulation of these cells in the elderly resulted in higher absolute numbers of functional anti-CMV cells in older subjects^19^.

Research interest in polyfunctional cells has gained a strong momentum in the last decade, as a critical role of polyfunctional T cells in immunological control has been shown in both human and animal studies^20^,^22^,^23^,^50^. Our study provides a novel observation that polyfunctional T-cell response is associated with higher level of anti-viral antibody. In our previous publication^42^, we have found that level of anti-CMV antibody is positively associated with viral load and negatively associates with T-cell receptor repertoire diversity. Thus it is intriguing to study if repertoire diversity is related to polyfunctionality. However, the appropriate definition of polyfunctionality and its relation to clinical outcomes remain poorly understood. T cells with early memory features such as IL-2 secretion and proliferation may be especially important in maintaining the antigen-specific T-cell population, but these cells are not capable of rapid perforin upregulation and efficient target cell killing. On the other hand, a definition of polyfunctionality without cytotoxic functions will not capture the complete picture of anti-viral cellular response. Our study indicates that different definitions of polyfunctionality are necessary and could lead to distinct relationships between polyfunctionality and total response size (Fig. 3). As a result, caution is needed when interpreting study results in the literature on T-cell polyfunctionality.

In summary, CD4 + and CD8 + CMV-pp65-specific T cells in older adults exhibit increasing polyfunctionality in association with increasing response size. Such changes in cytotoxic polyfunctionality maturation are positively related to the intensity of the humoral response. Our findings may not apply to all T-cell responses against other viruses and may not necessarily apply to other CMV viral proteins. However, our data support a model in which virus-specific CD4 + and CD8 + T cells synergistically contribute to the quantity and quality of the memory T-cell response during latent infection in aging. Further studies are required to determine whether CMV-specific T-cell polyfunctionality reflects the frequency and magnitude of prior episodes of viral reactivation, and whether it is associated with any significant clinical outcomes.

## Additional Information

**How to cite this article**: Chiu, Y.-L. *et al.* Cytotoxic polyfunctionality maturation of cytomegalovirus-pp65-specific CD4+ and CD8+ T-cell responses in older adults positively correlates with response size. *Sci. Rep.* **6**, 19227; doi: 10.1038/srep19227 (2016).

## Figures and Tables

**Table 1:** Close relationships among CMV IgG level, total pp65-specific T-cell response size, and cytotoxic polyfunctionality.

	Spearman R	95% confidence interval	p value
Correlation with total CMV-specific CD8 + T-cell response size			
CMV Ig level (IU/ml)	0.32	0.009 to 0.50	0.006*
Total CMV-specific CD4 + T-cell response size (%CD4 + )	0.35	0.10 to 0.55	0.005*
CMV-specific CD4 + IL-2 polyfunctionality (I + T + 2 + %CD4 + )	0.57	0.39 to 0.70	<0.001*
CMV-specific CD4 + cytotoxic polyfunctionality (T + I + C + P + %CD4 + )	0.24	−0.012 to 0.47	0.054
Correlation with total CMV-specific CD4 + T-cell size			
CMV Ig level (IU/ml)	0.23	0.029 to 0.44	0.049*
CMV-specific CD8 + IL-2 polyfunctionality (I + T + 2 + %CD8 + )	0.007	−0.18 to 0.31	0.56
CMV-specific CD8 + cytotoxic polyfunctionality (T + I + C + P + %CD8 + )	0.32	0.082 to 0.53	0.008*
Correlation with total CMV IgG level (IU/ml)			
CMV-specific CD8 + IL-2 polyfunctionality (I + T + 2 + %CD8 + )	0.21	−0.04 to 0.43	0.094
CMV-specific CD8 + cytotoxic polyfunctionality (T + I + C + P + %CD8 + )	0.34	0.15 to 0.55	0.001*
CMV-specific CD4 + IL-2 polyfunctionality (I + T + 2 + %CD4 + )	0.15	−0.10 to 0.38	0.146
CMV-specific CD4 + cytotoxic polyfunctionality (T + I + C + P + %CD4 + )	0.35	0.13 to 0.53	0.002*

Spearman’s rank-order correlation test was used to detect significant correlations between two parameters of interest. CMV-IgG level showed significant positive associations with CD4 + and CD8 + cytotoxic polyfunctional cell frequencies.
